# Aging-related changes in knee flexor muscle strength and cross-sectional area

**DOI:** 10.1097/MD.0000000000031104

**Published:** 2022-10-21

**Authors:** Soo Yeon Park, Kyoung Ho Yoon, Sung Hyun Hwang, Taeg Su Ko, Hee Sung Lee

**Affiliations:** a Department of Physical Education, Graduate School of Education, Yong In University, Yongin-si, Gyeongki-do, Republic of Korea; b Department of Orthopaedic Surgery, Kyung Hee University Hospital, Seoul, Republic of Korea; c Department of Medicine, Graduate School, Kyung Hee University, Seoul, Republic of Korea.

**Keywords:** cybex, dynapenia, isokinetic peak torque, muscle strength, sarcopenia

## Abstract

Weakening muscle strength around the knee tends to render it vulnerable to aging-related damage. This study aimed to examine the association between knee flexor muscle strength and its cross-sectional area (CSA). We also evaluated aging-related changes in flexor muscle strength and CSA. We retrospectively analyzed 252 patients with acute-onset knee pain (<1 month) between September 2006 and August 2009 in accordance with the Strengthening the Reporting of Observational studies in Epidemiology statement. The CSA of each knee flexor muscle (biceps femoris, sartorius, gracilis, semitendinosus (ST), and semimembranosus (SM)) was measured on magnetic resonance imaging axial images at the suprapatellar level. We evaluated flexor muscle strength (peak torque in N.m) using a Cybex dynamometer at 60°/second and 180°/second and its correlation with CSA. In total, 252 patients (mean age, 34.5 years; range, 11 to 66 years; 184 men and 68 women) were included in this study. No significant intergroup differences in demographic data such as sex or body mass index were found. Mean CSA was 605.4 mm^2^ for the SM, 444.7 mm^2^ for the biceps femoris, 282 mm^2^ for the sartorius, 55.4 mm^2^ for the ST, and 34.1 mm^2^ for the gracilis. Mean peak torques were 67.4 N.m and 52.7 N.m at 60°/second and 180°/second, respectively. CSA was positively correlated with flexion strengths of 60°/second (*R* = 0.363, *P* < .001) and 180°/second (*R* = 0.354, *P* < .001). Muscle strength was associated with CSA in all muscles but the gracilis (*R* = 0.056, *P* = .375). Flexion strength decreased significantly with aging from the thirties. Total CSA decreased with aging (*r* = −0.247, *P* < .001). The CSA of the biceps femoris, sartorius, SM, and ST decreased significantly, whereas that of the gracilis tended to decrease non-significantly with aging. Flexor muscle strength was associated with total muscle CSA on magnetic resonance imaging and the CSA of every muscle except the gracilis. Flexion strength decreased significantly with aging after the twenties, while total CSA decreased significantly with aging. The CSA of all flexor muscles decreased significantly with aging, whereas that of the gracilis decreased only slightly.

## 1. Introduction

As average age and life expectancy increase, the population of active patients with sports-related knee injuries who still maintain an active lifestyle is expected to increase across all age groups.^[1]^ Thus, understanding the muscles around the knee is important for patients, orthopedic physicians, and rehabilitation trainers. Weakening muscle strength around the knee tends to render it vulnerable to aging-related damage. Aging-related decreases in muscle size and strength are characteristic of the human body.^[2–4]^ This decline in muscle strength leads to decreased functional and living abilities and an increased risk of injury associated with common daily activities.^[5,6]^ Thus, a quantitative assessment of muscle strength around the knee joint is required. A correlation between muscle strength and muscle cross-sectional area (CSA) has also been reported,^[7–9]^ and muscle size is an important determining factor for muscular strength.^[10,11]^ However, the proportion of bending force according to muscle type and the effect of aging on muscle strength remain unclear.

Therefore, here we hypothesized that muscle strength is associated with muscle CSA on magnetic resonance imaging (MRI) and that the relationship between muscle strength and CSA differs among the flexor muscles. We also hypothesized that muscle strength and CSA decline with aging at rates that differ among the flexor muscles. To evaluate this hypothesis, we studied the relationship between the flexor muscle strength of the knee joint and flexor muscle CSA on MRI. We also compared the strength and CSA of each flexor muscle and evaluated the changes in flexor muscle strength and CSA on MRI with aging.

## 2. Methods

### 2.1. Patient details

We retrospectively analyzed 252 patients who visited the outpatient clinic with acute-onset knee pain (<1 month) between September 2006 and August 2009 in accordance with the Strengthening the Reporting of Observational studies in Epidemiology statement.^[12]^ The inclusion criteria were as follows: acute-onset knee pain within < 1 month of symptom onset; and use of both knee MRI and a Cybex evaluation. Patients were excluded from the study if they had chronic knee pain (≥1 month) or could not undergo the Cybex test because of persistent knee pain. The demographic data of the enrolled patients included age, sex, and body mass index. Patients were divided into five groups by age (10–19, 20–29, 30–39, 40–49, and ≤ 50 years).

### 2.2. MRI muscle quantification

**Figure 1.**
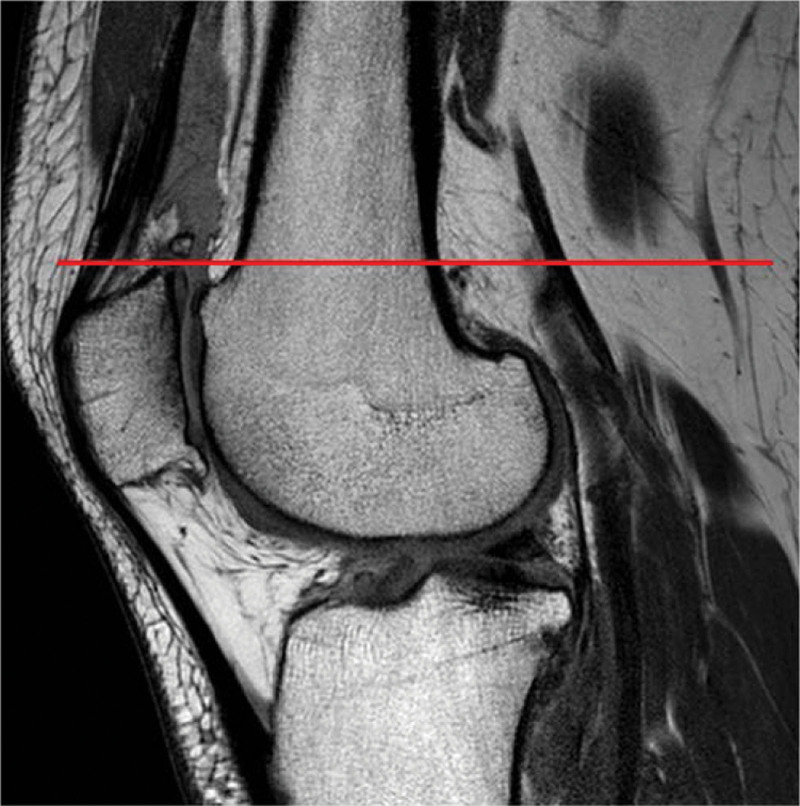
Measurement of flexor muscle cross-sectional area at the level of the patellar upper pole.

**Figure 2.**
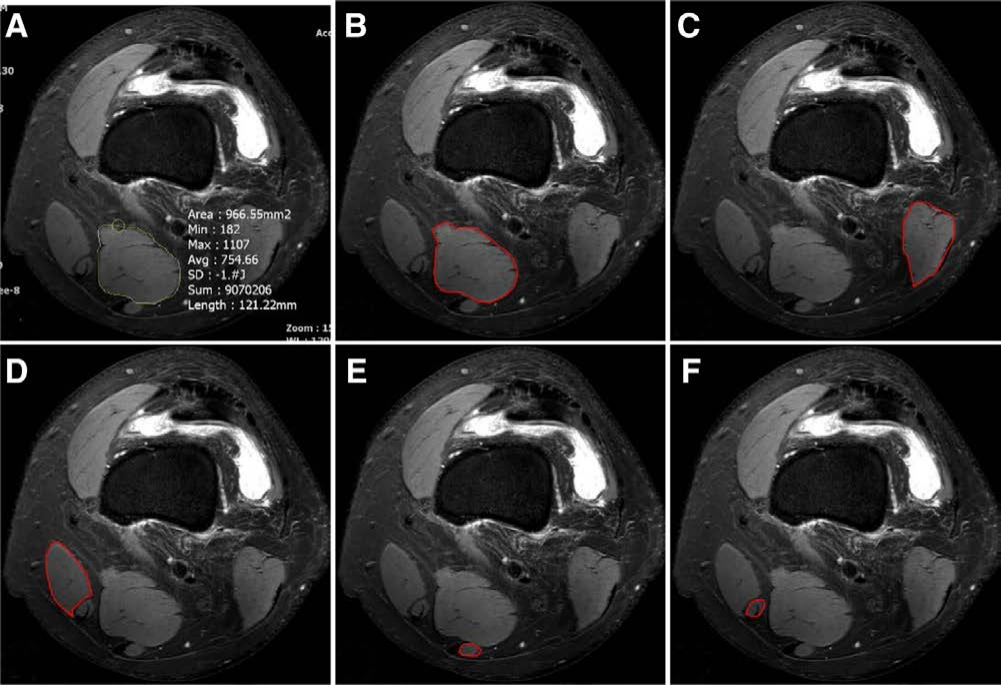
Cross-sectional area of the knee flexor muscles recorded using the INFINITT system. (A) The free-hand technique was used. (B) The SM, (C) biceps femoris, (D) sartorius, (E) ST, and (F) gracilis were recorded on the INFINITT system using a muscle outline. SM = semimembranosus, ST = semitendinosus.

All patients underwent MRI (3-T Achieva; Philips Medical Systems, Andover, MA, USA). Images were collected using a repetition time of 4349 ms, echo time of 30 ms, and slice thickness of 4 mm. A matrix size of 320 × 307 was used for all scans. We measured the knee flexor muscle CSA (biceps femoris, sartorius, gracilis, semitendinosus (ST), and semimembranosus (SM)) on an MRI axial view^[13]^ at the patellar upper pole (Fig. 1).^[14]^ It was approximately 87% of the femoral length (distance between the greater trochanter and lateral condyle of the femur; distal 0% to proximal 100%). The lining along the boundary of the muscle outline was recorded for the CSA of the knee flexor muscles on the INFINITT PACS (Infinitt Healthcare, Seoul, Korea) using the freehand technique (Fig. 2). The measurements were performed by a board-certified orthopedic sports medicine fellowship-trained surgeon (T.S.K.).

### 2.3. Knee flexor muscle isokinetic strength

Lower-extremity strength, especially of the knee flexors, is often studied because it correlates with functional activity.^[15,16]^ In this study, the Cybex test was performed at the 6-month follow-up in patients without pain to rule out muscle weakness due to pain. We measured flexor muscle strength (peak torque, N.m) using a Cybex isokinetic dynamometer (CYBEX International, Inc., Ronkonkoma, NY, USA) at 60°/second and 180°/second to objectively evaluate strength. The patients sat on the examination table with their upper body and thighs fixed. The Cybex workout axis coincided with the axis of knee flexion, while the lower-extremity axis was parallel to the dynamometer arm. Muscle power was recorded at 60°/second after four exercise repetitions. The test was repeated at 180°/second in the same manner. Measurements were performed twice at the two angular positions to determine the mean value.

### 2.4. Statistical analysis

Data are reported as mean ± standard deviation. SPSS software ver. 21.0 (SPSS Inc., Chicago, IL) was used for the analyses. Correlations between muscle strength and CSA were analyzed using the Pearson’s correlation coefficient test. Muscle strength and CSA by age were assessed using one-way analysis of variance. Values of *P *< .05 were considered statistically significant.

## 3. Results

### 3.1. Patient demographics

In total, 252 patients (mean age, 34.5 years; range, 11–66 years; 184 men and 68 women) were included in this study. The demographic data of the five groups are summarized in Table 1. No significant intergroup differences were found in demographic data such as sex or body mass index.

**Table 1:** Number of patients in each age group.

Age group	10 to 19 (n = 20)	20 to 29 (n = 93)	30 to 39 (n = 56)	40 to 49 (n = 47)	≤50 (n = 36)	*P* value
**Sex (male:female**)	15:5	68:25	45:11	31:16	25:11	.564
**BMI (kg/m^2^**)	23.0 ± 2.3	23.3 ± 3.3	24.0 ± 3.9	22.9 ± 2.9	23.7 ± 2.3	.470

BMI = body mass index.

### 3.2. Muscle strength and CSA

The SM muscle had the highest CSA. The mean values were SM, 605.4 mm^2^; biceps femoris, 444.7 mm^2^; sartorius, 282 mm^2^; ST, 55.4 mm^2^; and gracilis, 34.1 mm^2^ (Table 2). The mean peak torques were 67.4 N.m at 60°/second and 52.7 N.m at 180°/second (Table 3).

**Table 2:** Mean cross-sectional area of the knee flexor muscles.

	SM	Biceps femoris	Sartorius	ST	Gracilis	Total
**Mean CSA (mm^2^**)	605.4 ± 265.8	444.7 ± 162.5	282.0 ± 110.3	55.4 ± 45.7	34.1 ± 27.2	1421.7 ± 439.7

CSA = cross-sectional area, SM = semimembranosus, ST = semitendinosus.

**Table 3:** Mean peak torque at 60°/second and 180°/second.

	60°/second	180°/second
**Mean peak torque (N.m**)	67.4 ± 26.9	52.7 ± 20.6

**Figure 3.**
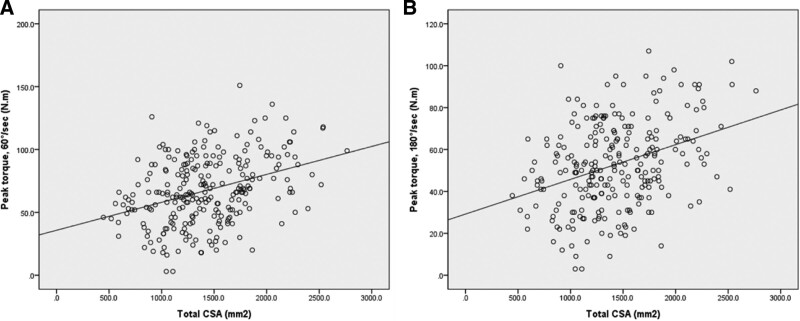
Correlation between isokinetic peak torque and total cross-sectional area of the knee flexor muscles. (A) 60°/second, *R* = 0.363, *P* < .001; (B) 180°/second, *R* = 0.354, *P* < .001.

**Figure 4.**
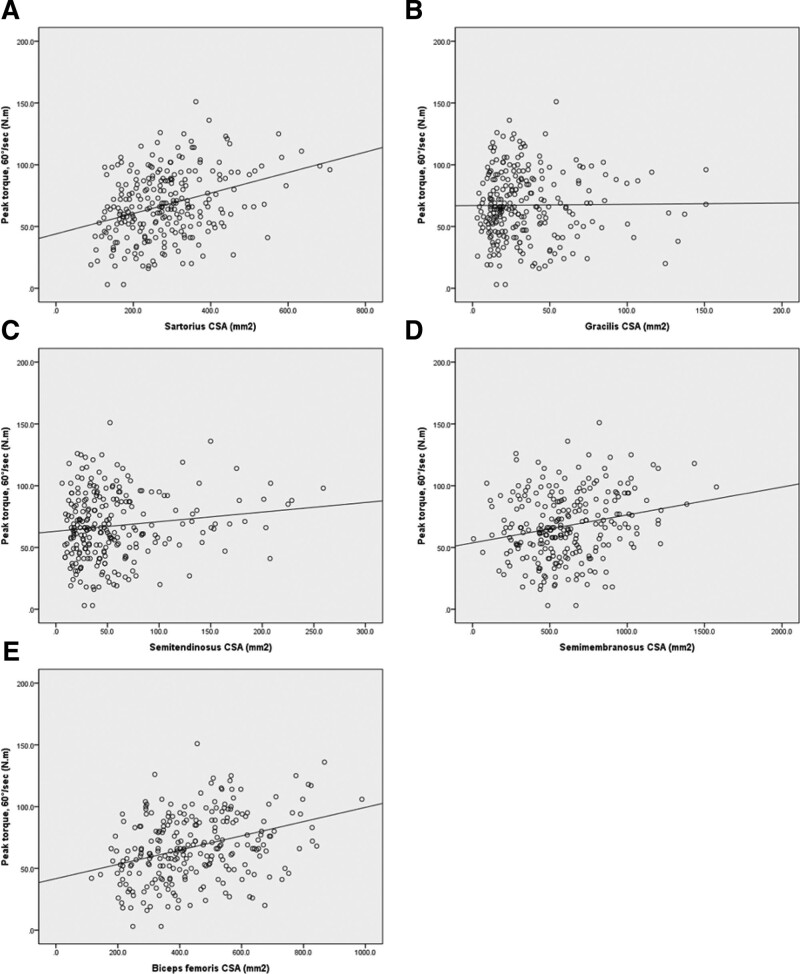
Correlation between isokinetic peak torque at 60°/second and cross-sectional area of the knee flexor muscles. (A) sartorius, *R* = 0.339, *P* < .001; (B) gracilis, *R* = 0.010, *P* = .877; (C) ST, *R* = 0.132, *P* = .037; (D) SM, *R* = 0.223, *P* < .001; (E) biceps femoris, *R* = 0.348, *P* < .001. SM = semimembranosus, ST = semitendinosus.

**Figure 5.**
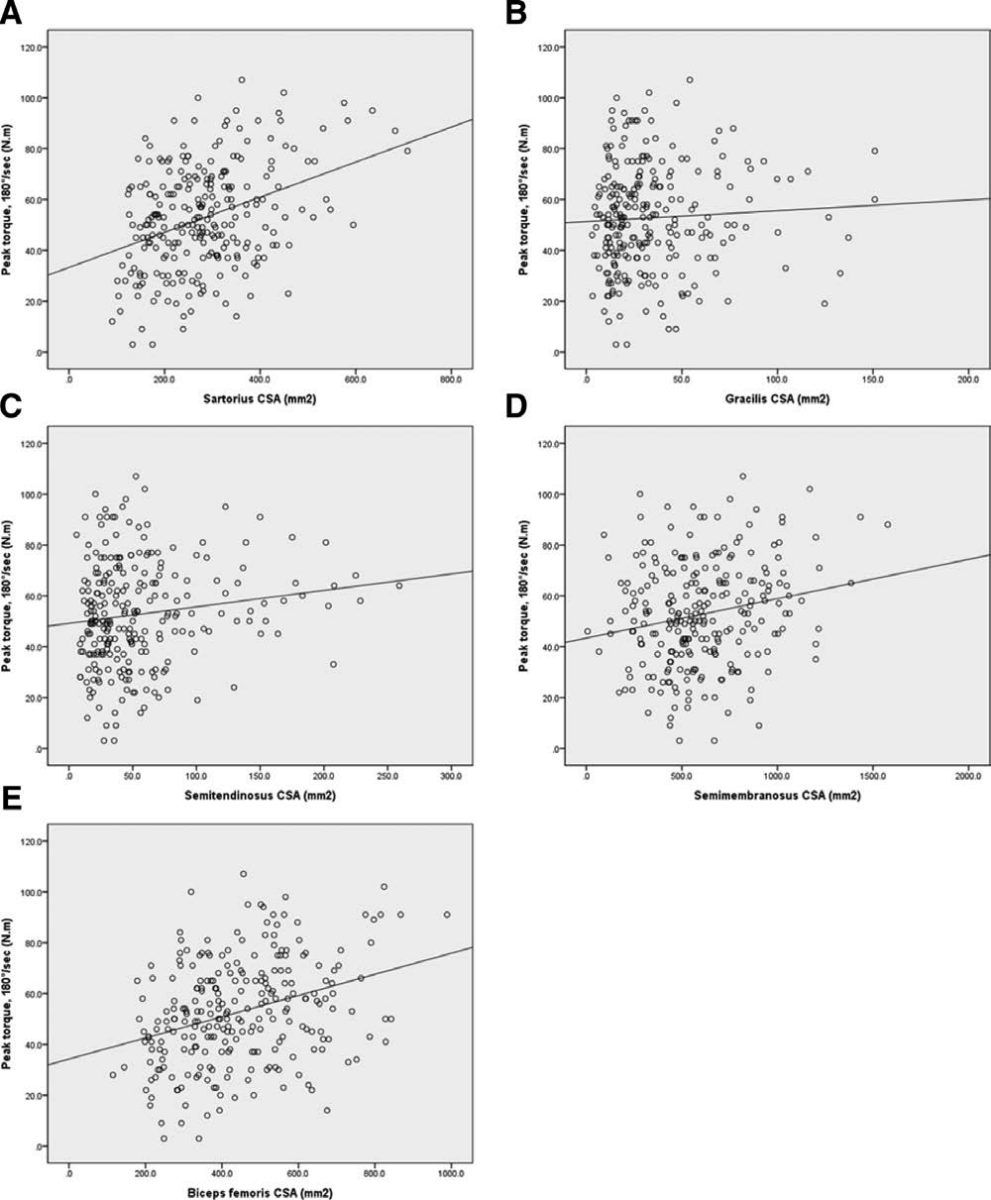
Correlation between isokinetic peak torque at 180°/second and cross-sectional area of the knee flexor muscles. (A) sartorius, *R* = 0.369, *P* < .001; (B) gracilis, *R* = 0.056, *P* = .375; (C) ST, *R* = 0.144, *P* = .022; (D) SM, *R* = 0.200, *P* = .001; (E) biceps femoris, *R* = 0.329, *P* < .001. SM = semimembranosus, ST = semitendinosus.

The total CSA was correlated with muscle strength at 60°/second (*R* = 0.363, *P* < .001) and 180°/second (*R* = 0.354, *P* < .001) (Fig. 3). All CSA values correlated with muscle strength at 60°/second and 180°/second with the exception of the gracilis muscle (*R* = 0.010, *P* = .877 at 60°/second; *R* = 0.056, *P* = .375 at 180°/second) (Figs. 4 and 5).

### 3.3. Muscle strength and CSA with aging

**Figure 6.**
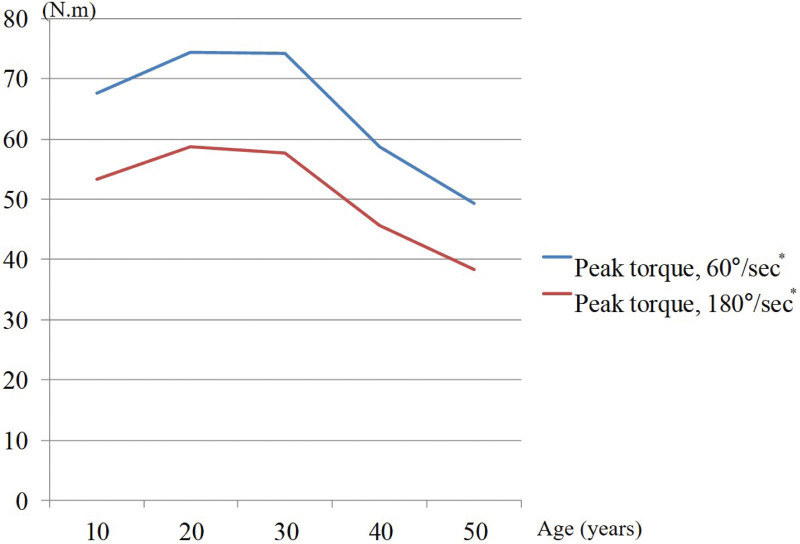
Changes in isokinetic peak torque with aging. Asterisks indicate significantly different values (*P* < .05).

**Figure 7.**
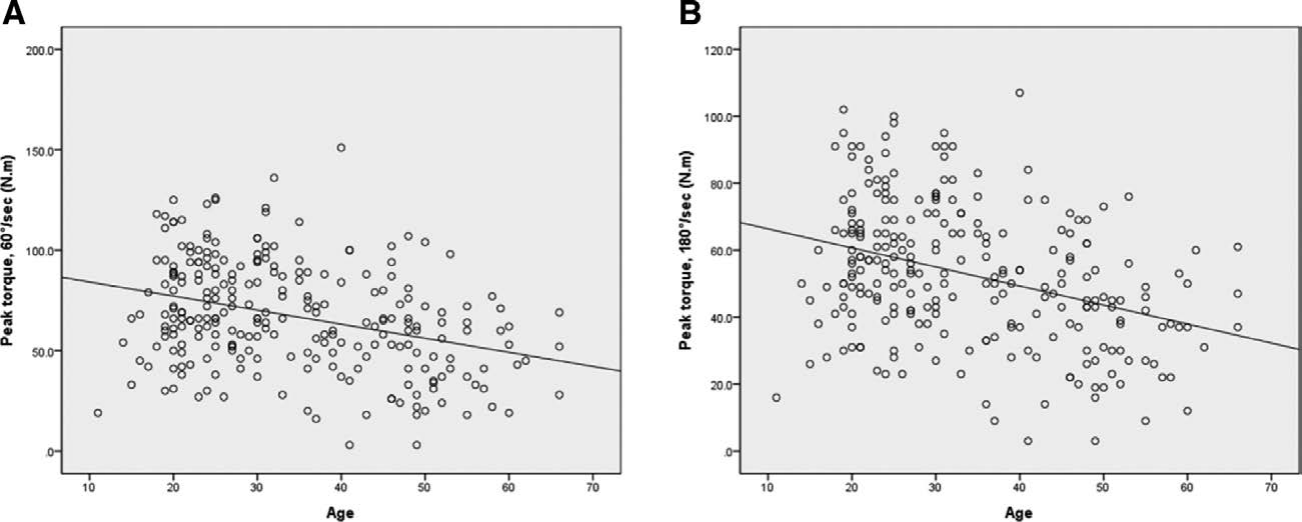
Correlation between isokinetic peak torque and age. (A) 60°/second, *r* = −0.332, *P* < .001; (B) 180°/second, *r* = −0.353, *P* < .001.

Muscle strength of the knee joint started to decrease in patients in their twenties and decreased significantly from their thirties. Muscle strength was negatively correlated with age at 60°/second (*r* = −0.332, *P* < .001) and 180°/second (*r* = −0.353, *P* < .001) (Figs. 6 and 7).

**Figure 8.**
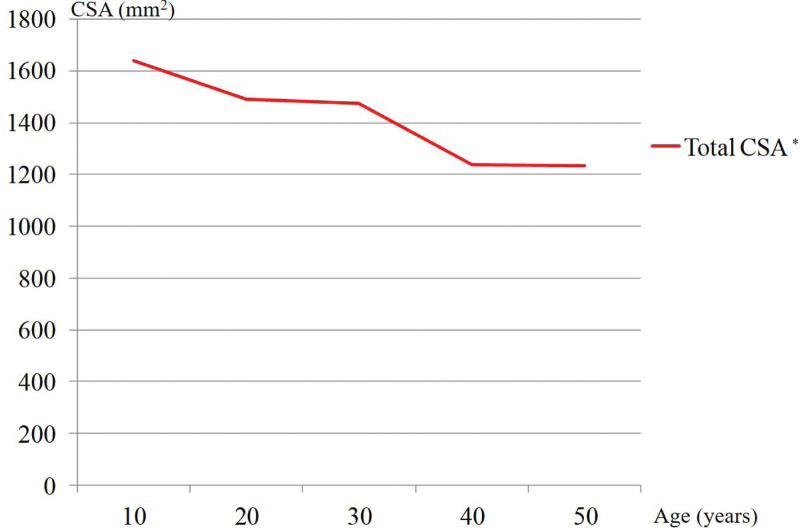
Changes in total cross-sectional area with aging. Asterisk indicates significantly different values (*P* < .05).

**Figure 9.**
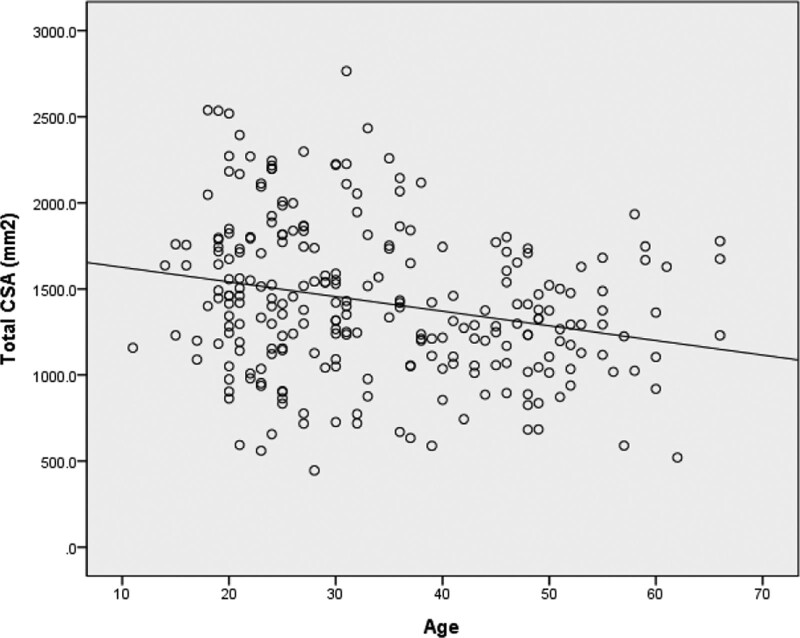
Relationship between total cross-sectional area and age. *R* = 0.247, *P* < .001.

**Figure 10.**
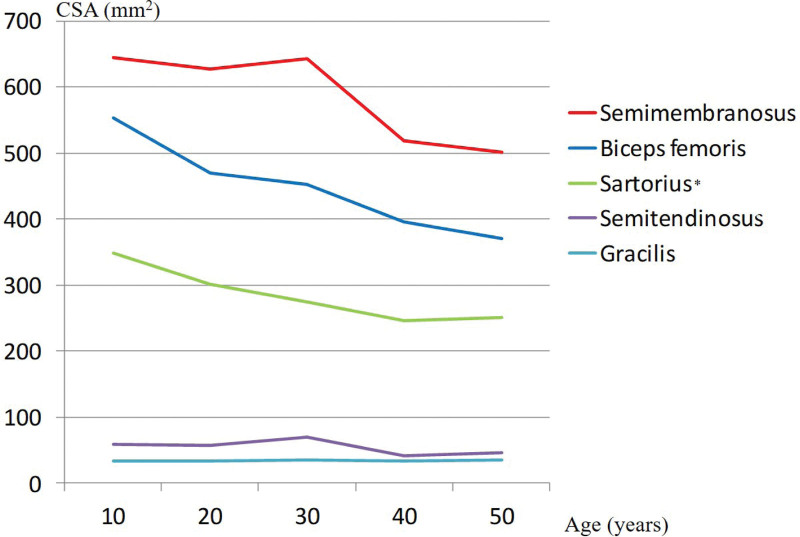
Changes in each cross-sectional area with aging. Asterisk indicates were significantly different values (*P* < .05).

Total CSA decreased significantly with aging. The total CSA was negatively correlated with age (*r* = −0.247, *P* < .001) (Figs. 8 and 9). All CSA values decreased significantly with aging except for that of the gracilis (Fig. 10). The muscle CSA reduction rates were as follows: biceps femoris, 33.1%; sartorius, 28%; SM, 22.3%; ST, 21.9%; and gracilis, 0.6% (Table 4).

**Table 4:** Reduction rate of each cross-sectional area with aging.

Muscle	Reduction rate (%)	*P* value
**Biceps femoris**	33.1	<.001
**Sartorius**	28.0	.001
**SM**	22.3	.018
**ST**	21.9	.023
**Gracilis**	0.6	.995

SM = semimembranosus, ST = semitendinosus.

## 4. Discussion

This study measured knee flexor muscle volume on MRI and determined the maximum flexion strength values. Our initial hypothesis – muscle strength and CSA decline with aging at rates that differ among the flexor muscles – was confirmed. The results of our study are consistent with those of previous studies showing that aging leads to marked changes in muscle volume and strength.^[2–4]^

Age-related maximum intensity is known to occur at 20 to 30 years.^[17,18]^ Karsten Keller et al^[19]^ reported that the decline in muscle strength of people younger than versus older than 40 years was 16.6% to 40.9%. Total CSA, with the exception of that of the gracilis, and knee flexor strength decreased in older versus younger adults. Bruce et al^[20]^ reported that the regression line between muscle strength and CSA cannot have a true intercept, because if muscle CSA equals zero, there must be no force. Akagi et al^[7]^ reported that muscle volume is a major determinant of the joint torque in each knee joint muscle group. Our results showed that all flexor muscle CSA values (except that of the gracilis) were correlated with flexion power and that CSA decreased with aging. The mean CSA of the gracilis was 34.1 mm^2^. Therefore, a thin CSA did not affect knee flexion power, and no change with aging was observed.

Aagaard et al^[13]^ reported that training-induced changes in muscle fiber CSA differ among fiber types; i.e., type II fibers, which are recruited as the level of force increases, show more prominent hypertrophy than type I fibers. Johnson et al^[21]^ reported that the fiber-type composition is more or less specific to the muscles and that there is a greater difference in the percentage of type II fibers between the anterior thigh and posterior muscle groups. In an animal study, Ariano et al^[22]^ discovered more type II muscle fibers in the SM, and that the ST had nearly 0% type II muscle fibers. Johnson et al^[21]^ reported that a greater number of type II muscle fibers are present in the sartorius and biceps femoris in humans.

Knee flexor muscle volume and strength decreased significantly in older versus younger adults.^[23–25]^ Other age-related neuromuscular changes that have also been implicated in strength deficits include infiltration of intramuscular fat, increased connective tissue, reduced contractile tissue, reduced neural drive, neuromuscular junction changes, increased antagonist muscle co-activation, decreased muscle fiber–specific tension, and preferential atrophy of type II muscle fibers.^[26,27]^ Thus, the lack of a decrease in gracilis volume can be explained by its fewer type II muscle fibers.

The loss of knee flexor strength following the harvest of the ST tendon and the gracilis tendon in anterior cruciate ligament reconstruction has been described in the literature.^[28,29]^ The muscle and tendon morphologic features of the ST and gracilis are substantially altered after tendon harvest, and knee muscle strength is gradually restored to approximately 80% of the uninjured leg even 1 year after surgery, but not completely.^[30,31]^ Taken together, our results suggest that anterior cruciate ligament reconstruction using auto-tendon graft in elderly patients with decreased flexor muscle volume and muscle strength may cause additional weakness in knee flexion strength.

This study has some limitations. First, despite measurements in patients with acute-onset knee pain, the results of the present study are probably not applicable to the general population without knee pain and with different physical activity levels. Second, there may be concern about muscle atrophy on MRI evaluations; however, since patients were excluded if they had acute-onset or chronic knee pain, muscle atrophy was likely minimal. Third, there may be sex-related differences in muscle strength or CSA within age groups; however, these differences were not analyzed in this study. Fourth, we checked the CSA on the knee MRI axial view at the level of the patellar upper pole and evaluated some of the gracilis and ST muscles at the tendinous level. This is a distal level of the femur; therefore, it is possible that knee flexor muscle strength was not properly reflected.

## 5. Conclusions

Based on currently available literature, this article provides a reasonably comprehensive analysis of aging-related changes in knee flexors. Flexor muscle strength was associated with total muscle CSA on MRI and the CSA of each muscle except the gracilis. Flexion strength decreased significantly with aging after the twenties, while total CSA decreased significantly with aging. The CSA of the biceps femoris, sartorius, SM, and ST decreased significantly with aging, whereas that of the gracilis decreased only slightly. These results will be useful for orthopedic physicians and rehabilitation trainers when discussing treatment plans considering the age of patients with knee injuries.

## Author contributions

SYP contributed to the study conception and design, data analysis and interpretation, and manuscript drafting and revision. KHY contributed to the study conception and design; data acquisition, analysis, and interpretation; and manuscript drafting and revision. TSK and SHH contributed to the data acquisition and interpretation. HSL contributed to the study conception and design, data analysis and interpretation, manuscript drafting and revision, and final article approval. All authors contributed to and approved the final manuscript.

**Conceptualization:** Soo Yeon Park, Kyoung Ho Yoon, Hee Sung Lee.

**Data curation:** Soo Yeon Park, Taeg Su Ko, Hee Sung Lee.

**Formal analysis:** Soo Yeon Park, Kyoung Ho Yoon, Taeg Su Ko, Hee Sung Lee.

**Investigation:** Soo Yeon Park, Kyoung Ho Yoon, Sung Hyun Hwang, Taeg Su Ko, Hee Sung Lee.

**Methodology:** Soo Yeon Park, Sung Hyun Hwang, Hee Sung Lee.

**Project administration:** Soo Yeon Park, Kyoung Ho Yoon, Hee Sung Lee.

**Resources:** Sung Hyun Hwang, Taeg Su Ko.

**Software:** Sung Hyun Hwang, Taeg Su Ko.

**Supervision:** Kyoung Ho Yoon, Hee Sung Lee.

**Validation:** Kyoung Ho Yoon, Hee Sung Lee.

**Visualization:** Kyoung Ho Yoon, Hee Sung Lee.

**Writing – original draft:** Soo Yeon Park, Hee Sung Lee.

**Writing – review & editing:** Soo Yeon Park, Hee Sung Lee.
